# Internet-based individually versus group guided self-help treatment for social anxiety disorder: protocol of a randomized controlled trial

**DOI:** 10.1186/1471-244X-14-115

**Published:** 2014-04-15

**Authors:** Ava Schulz, Timo Stolz, Thomas Berger

**Affiliations:** 1 Department of Clinical Psychology and Psychotherapy, University of Bern, Bern, Switzerland

**Keywords:** Social anxiety disorder, SAD, Social phobia, Randomized controlled trial (RCT), Internet-based treatment, Self-help, Guided self-help, Group guided self-help

## Abstract

**Background:**

Social anxiety disorder (SAD) is one of the most common mental disorders and causes subjective suffering and economic burden worldwide. Although effective treatments are available, a lot of cases go untreated. Internet-based self-help is a low-threshold and flexible treatment alternative for SAD. Various studies have already shown that internet-based self-help can be effective to reduce social phobic symptoms significantly. Most of the interventions tested include therapist support, whereas the role of peer support within internet-based self-help has not yet been fully understood. There is evidence suggesting that patients’ mutual exchange via integrated discussion forums can increase the efficacy of internet-based treatments. This study aims at investigating the added value of therapist-guided group support on the treatment outcome of internet-based self-help for SAD.

**Methods/design:**

The study is conducted as a randomized controlled trial. A total of 150 adults with a diagnosis of SAD are randomly assigned to either a waiting-list control group or one of the active conditions. The participants in the two active conditions use the same internet-based self-help program, either with individual support by a psychologist or therapist-guided group support. In the group guided condition, participants can communicate with each other via an integrated, protected discussion forum. Subjects are recruited via topic related websites and links; diagnostic status will be assessed with a telephone interview. The primary outcome variables are symptoms of SAD and diagnostic status after the intervention. Secondary endpoints are general symptomology, depression, quality of life, as well as the primary outcome variables 6 months later. Furthermore, process variables such as group processes, the change in symptoms and working alliance will be studied.

**Discussion:**

The results of this study should indicate whether group-guided support could enhance the efficacy of an internet-based self-help treatment for SAD. This novel treatment format, if shown effective, could represent a cost-effective option and could further be modified to treat other conditions, as well.

**Trial registration:**

ISRCTN75894275

## Background

Social anxiety disorder (SAD) is the third most common mental disorder after depression and alcohol dependency and is considered the single most prevalent anxiety disorder [1]. Characteristic for this disorder is the individual’s extreme fear that others might judge their behavior or physical symptoms as embarrassing [2]. The resulting high psychological suffering is furthermore the reason for high economic costs, such as low productivity at work, loss of working hours and under-qualification [1,3]. Without professional intervention, SAD tends to be a chronic condition [4]. Although effective psychological treatments and drug therapies are available [5], only a small proportion of those in need receive empirically-supported treatment [6]. This has several causes: In addition to factors that are valid for psychological disorders in general (such as fear of stigmatization, financial strains or the lack of qualified therapists in the immediate vicinity), the very nature of SAD can prevent individuals from seeking professional advice [1,7]. Against this backdrop, the current challenge is to make low-threshold, easily accessible treatment alternatives available to the high percentage of untreated individuals suffering from SAD. Internet-based treatments with their high level of anonymity and privacy and their easy accessibility seem to fit this profile.

Over recent years, the number of controlled trials evaluating Internet-based interventions has grown rapidly. By now, more than 100 controlled studies evaluating Internet-based treatments in the field of anxiety disorders, depression and other conditions have shown promising results [8-12]. Particularly in the treatment of anxiety disorders, independent replications have shown moderate to large effect sizes in comparison with control groups [9,10,12,13]. Most of this promising body of evidence comes from studies evaluating Internet-based guided self-help treatments, in which the presentation of a Web-based self-help program is combined with minimal but regular therapist contact via e-mail. Emerging evidence from meta-analyses (e.g. [12]) and from a reanalysis of data across trials [14] suggests a superiority of guided versus unguided self-help treatments in terms of efficacy, adherence to treatment and drop-out rates. However, therapist guidance does not seem to be needed in some disorders and under some conditions. For instance, in the treatment of SAD, good outcomes have been reported for unguided treatments if a proper diagnosis has been established [15,16]. SAD is among the most researched disorders in the field of internet-based treatments and it is a condition for which strong empirical support for the efficacy of Internet-based treatments exist [17]. However, there is room for optimizing and improving Internet-based treatments for SAD, as still a considerable number of patients do not benefit from or recover fully after an Internet-based treatment (e.g. [15]).

The general aim of this randomized controlled trial is to evaluate an internet-based treatment format which takes into account that in Internet-based treatments participants can also enter into an exchange with each other. Several prior studies on Internet-based treatments for SAD have included the possibility of peer support in moderated or unmoderated discussion forums with encouraging results (e.g. [15,18,19]). These forums are usually conceptualized and used as an additional option complementing the main treatment components such as the web-based self-help guide and individual therapist guidance. There is some initial evidence across studies that the additional peer support might increase the benefits of Internet treatments for SAD. For instance, Titov and colleagues could improve small within-group effect sizes for an unguided Internet-based self-help treatment for SAD [20] by adding a clinician-moderated online discussion forum [18]. Another study found comparable effects for an Internet-based guided self-help program for SAD and the same self-help guide delivered in the form of bibliotherapy with no therapist support but with access to an online discussion forum [19]. The use of an online discussion alone, without self-help guide, does not seem to lead to substantial changes in social anxiety [21].

Our own experience with integrated discussion forums is that many participants value the possibility to share information and experiences with other participants as one of the most helpful elements of the whole treatment package [22,23]. Moreover, qualitative analysis of the content of the discussions in our forums showed that therapeutic mechanisms of change, that are known from research on group psychotherapies, may come into play (e.g., normalization, altruism, instillation of hope, development of socializing techniques, interpersonal learning, imitative behavior, cohesiveness [24]). But there are also problems related to the use of discussion forums in Internet-based treatments. A practical problem is that participants usually do not start exactly at the same time with such treatments, they are therefore at different points in the treatment, and they may have different issues to discuss. Another problem is that while usually all participants read the messages in the discussion forums, only about half of them also post their own messages [15].

Reasons for not actively participating are the optional nature of the forums and, as indicated by many participants, the fact that they do not want to write to a large group of anonymous users [24]. These problems are addressed in this study in an Internet-based group guided self-help treatment in which small therapist-guided groups of participants start simultaneously, introduce themselves to each other, and then conjointly work their way through the Internet-based self-help guide. It should be noted that the Internet-based group treatment is different from group therapy in face-to-face settings as the main component of the intervention is a self-help program. Regarding group therapies for SAD, evidence suggests that the group situation may be too threatening for social phobics, resulting in individuals inverting or “shutting down” and gaining less in group settings than in individual settings [25]. In the Internet-based setting, the asynchronous, distant communication with other group members may not provoke overwhelming anxiety symptoms and allows participants to concentrate on the content of the treatment.

### Objective and research questions

The primary aim of this study is to evaluate whether the newly developed group guided self-help treatment for SAD is effective in reducing social anxiety symptoms when compared to a waiting-list control group and an individually guided self-help treatment. It is expected that the active intervention groups will be superior to the waiting-list control group with regard to primary social anxiety measures and secondary outcomes such as depression, general symptomatology, interpersonal problems and quality of life. It is further expected that the group guided self-help treatment will be superior to the individually guided self-help treatment in terms of adherence to treatment, treatment satisfaction and cost as estimated from the amount of therapist time required for each client.

## Methods/design

### Study design

This study is conducted as a randomized controlled trial. Participants are randomly assigned to either of the active conditions or the control condition. Main component of the active conditions is an internet-based self-help program, which will either include individual support by a psychologist or therapist-guided peer support in groups of six participants.

### Ethical approval

This study has been approved on 14/06/2013 by the Cantonal Ethics Committee Bern (ref. nr. KEK-BE: 062/13).

### Participants

A total of 150 fulfilling the diagnostic criteria of SAD are being recruited, with 60 participants assigned to each of the active conditions and 30 participants to the waiting-list control group. Participants are recruited in Switzerland, Austria and Germany through a study website and postings in Internet forums.

### Inclusion criteria

1 The patient is at least 18 years of age.

2 The patient provides a written informed consent.

3 The patient has access to a computer with internet connection.

4 The patient has sufficient German language skills to benefit from the program and provide usable data through questionnaires and interviews.

5 The patient exceeds pre-defined cut-off scores in one of the social anxiety measures.

6 The patient meets the diagnostic criteria for SAD according to the diagnostic telephone interview.

7 The patient agrees to undergo no other psychological treatment for the duration of the study. The patient agrees to undergo no other psychological treatment when joining the study (Patients are asked for report, if they do so later on while taking part in the study. In this case, this patients’ data would be excluded from evaluation.)

### Exclusion criteria

1 The patient has active suicidal plans (according to the suicide item of the BDI or the diagnostic telephone interview). These individuals are referred to a local psychiatrist or psychotherapist.

2 The patient has a history of psychotic or bipolar disorders.

3 Prescribed medication for anxiety or depression only leads to exclusion if the dosage has been changed during the last month prior to the study.

### Randomization

After checking the inclusion criteria, a weighted randomization procedure is used in which 60 participants are assigned to each of the active treatment conditions and 30 participants to the waiting-list control group. The allocation list is produced by a computerized random number generator operated by an independent researcher and is unknown to the investigators.

### Description of the interventions

a) Internet-based individually guided self-help without peer support:

The main component of the 12-week treatment is a self-help program, which has been successfully evaluated in previous studies [15,23,26,27]. The theoretical background of this program is based on Clark & Wells’ well-established cognitive-behavioral treatment model [28]. The program contains eight text-based sessions, including several exercises (such as attention training) and diaries (e.g. diaries to identify and challenge dysfunctional assumptions). Participants are asked to work trough one session per week, repeat the exercises and fill in the diaries. One session can be completed in approximately 60 minutes. Table 1 shows an overview of the content. While working with the program, the participants receive individual support by psychologists via an integrated email function of the platform. The psychologists contact the participants on a weekly basis in order to provide structure and reinforce program use. The patients can use the email function to contact the psychologists whenever they want to. Supervision of the supporting psychologists is provided by the study direction. This intervention does not include any possibility of group-guided support (e.g. in a discussion forum).

b) Internet-based group guided self-help treatment:

Participants in the second active condition use the same internet-based self-help program as described above. In contrast, they will not be supported individually, but within groups of six participants. All members of one group start the intervention at the same time and use an integrated, protected discussion forum to communicate with each other under the guidance of a psychologist. At the beginning, the group members and the supporting professional introduce themselves. The group members are encouraged to share their experiences, problems and questions with each other. The messages posted in the forum are accessible to every group member and the psychologist, as well. Analogous to the first treatment condition, the psychologists contact the group on a weekly basis. The psychologists’ short messages aim at providing the group with structure and motivation.

The participants are asked to choose a pseudonym instead of their real name for the digital communication, so as to protect their privacy. The supporting psychologists supervise adherence to this guideline and will delete real names.

**Table 1:** **Content of the internet-based self-help program for SAD**[29]


Session 1: Motivational enhancement	Reasons to initiate change, definition of goals, recording of difficult social situations
Session 2: Psychoeducation	Information on SAD and its maintaining processes such as negative beliefs, self-focused attention and safety behaviors
	Development of an individual model for SAD
Session 3: Cognitive Restructuring	Identification and modification of dysfunctional assumptions using a thought record
Session 4: Self focused attention	Various exercises to reduce self-focused attention, e.g. short behavioral experiments
Session 5: Behavioral experiments	Planning and implementation of in vivo exposures
Session 6: Summary and Repetition	Summary of the key elements of the treatment with emphasis on the importance of repeated practice (e.g. in vivo exposure)
Session 7: Healthy lifestyle and problem solving	Information on healthy lifestyle behavior (physical exercise and nutrition)
	Development of problem solving skills
Session 8: Relapse prevention	Strategies for maintaining the skills learned, preparation for possible relapse

### Procedure

Patients are randomly assigned to either one of the active conditions or the waiting-list control condition. The patients in the active conditions use the program either with individual or group-guided support. The waiting-list control group gets to use the program 12 weeks later. All three groups are assessed at baseline (t0), immediately after completing the program (t1; 12 weeks) and at follow-up six months after randomization (t2). Additionally, participants in both of the active conditions complete self-report measures of SAD (Social Phobia Scale & Social Interaction Anxiety Scale; [30]) every two weeks. Other process measures are included according to the treatment condition (Working Alliance Inventory [31] for the group with individual support; Group Questionnaire [32], for the group-guided condition).

### Measurements

For an overview of assessment at baseline, 12-week-posttreatment and 6-month follow-up see Table 2.

**Table 2:** Measurements and time of assessment

**Instrument**	**Abbreviation**	**Aim**	**Time of assessment**
**Clinician administered**			
Structured Clinical Interview for DSM-IV Axis I Disorders	SCID-I	DSM-IV Axis I disorders	Pre, post (12 weeks), follow-up (6 moths)
**Self-report ratings**			
Primary Outcome Measures			
Social Phobia Scale	SPS	Symptoms of SAD	Pre, post, follow-up
Social Interaction Anxiety Scale	SIAS		
Secondary Outcome Measures			
Beck Depression Inventory	BDI-II	Symptoms of depression	Pre, post, follow-up
Brief Symptom Inventory	BSI	Psychiatric symptoms	Pre, post, follow-up
Inventory of Interpersonal Problems	IIP-64	Interpersonal difficulties	Pre, post, follow-up
SF-12 Health Survey (Quality of Life)	SF-12	Quality of Life	Pre, post, follow-up
Client Satisfaction Questionnaire	CSQ-8	Client satisfaction	Post
	ZUF-8		

### Primary outcome measures

#### Symptoms of SAD and diagnostic status

Social phobic symptoms and diagnostic status at post-treatment are the main outcome variables studied. Symptoms of SAD will be measured with self-report questionnaires using the Social Phobia Scale and the Social Interaction Anxiety Scale (SPS & SIAS; [30]). These two companion measures assess fears of being judged by others during daily activities (SPS) and more general fears in social interaction (SIAS), respectively. They have been found to be valid, reliable and useful for clinical and research purposes [33]. The patients’ diagnostic status will be assessed with a telephone interview conducted by trained raters and using the Structured Clinical Interview for DSM-IV (SKID-I [34]).

### Secondary outcome measures

#### Beck depression inventory II

To account for the high comorbidity of social phobia and depression, clients are asked to complete the German version of the Beck Depression Inventory II [35]. The BDI II is one of the most widely used self-report measures to assess depression in research in clinical practice. It consists of 21 items to be rated on a 4-point Likert-scale and has shown robust psychometric properties for internet delivery with SAD clients [15].

#### Brief symptom inventory

As a measurement of psychiatric symptoms, participants complete the German version of the Brief Symptom Inventory (BSI) [36]. The BSI assesses psychological distress on 9 dimensions such as anxiety, insecurity in social situations, depressiveness and compulsivity. The participants are asked to rate the occurrence of symptoms within the last week. Due to this restriction, the BSI is sensitive to change. As an economic screening instrument with robust psychometric properties, this inventory is commonly administered to detect pre-post changes [37].

#### Inventory of interpersonal problems

The Inventory of Interpersonal Problems (IIP) is used to identify sources of relational distress [38]. This self-report measure is designed to assess difficulties in interpersonal behavior on eight factor-analytically derived dimensions (domineering, vindictive, cold, socially avoidant, nonassertive, exploitable, overly nurturant, intrusive). The resulting T-scores of these subscales can be understood within the “interpersonal circumplex” model of personality [39]. The IIP has shown adequate psychometric properties [40].

#### Short-form health survey-12 (SF-12)

The SF-12 Health Survey is a condensed version of the Short Form Health Survey (SF-36) measuring physical and mental health on two subscales. Participants are asked to report the presence and severity of physical and mental problems over the course of the last 4 weeks. Its 12 items cover pain, psychological problems as well as impairments in everyday life. Because of its good re-test reliability and brevity, the SF-12 is widely used as an estimate of general quality of life [41].

#### Client satisfaction questionnaire (ZUF-8)

In order to learn more about the acceptance of web-based interventions, global client satisfaction is assessed at post-treatment using the Client Satisfaction Questionnaire (ZUF-8) [42]. The ZUF-8 is a translation of the Client Satisfaction Questionnaire [43] which was originally developed to measure satisfaction with inpatient treatment. We adapted the German version in this study to assess contentment with the web-based self-help treatment.

#### Adherence to treatment /completion rates

Adherence to treatment and completion rates as one of the major problems of internet-based self-help will be assessed, as well. More specifically, lessons completed, time spent in the self-help program and usage of the different types of support will be analyzed to identify potential mediating effects.

### Process measures

#### Change in social phobic symptoms

During the intervention, participants in the active conditions are asked to rate their symptoms of SAD every two weeks (SPS & SIAS).

#### Working alliance and group processes

Because the role of the therapeutic relationship within internet-based intervention has yet to be fully understood, patients in the condition with individual support fill out the Working Alliance Inventory (WAI [31]). This instrument comprises bond, goal and task aspects of the working alliance and shows an adequate reliability. Consistent with previous research on internet-based interventions, a modified version of the WAI is administered to account for the self-help character of the treatment [29].

Correspondingly, participants in the group guided self-help condition are asked to report their experiences with the Group Questionnaire (GQ-D [44]). The GQ-D assesses helpful aspects of relationships within small groups, hereby enabling us to further examine group processes in web-based interventions.

### Power and sample size calculation

The main endpoints in this study are the reduction of social phobic symptoms and the patients’ diagnostic status. Regarding differences between the two active conditions, we want to be able to demonstrate standardized effect sizes (Cohen’s d) of 0.35, since smaller effects are considered clinically insignificant. At an α error level of 0.05, a statistical power (1-Beta) of 0.80, and a correlation between pre- and post-measurements found in our previous studies, 60 participants are needed in each of the two active conditions. Based on earlier findings, we expect large differences in the comparison of the active conditions and the waiting-list control. Power analysis shows that a sample size of 30 in the waiting-list control group will be sufficient to show effect sizes of this magnitude.

### Analysis

Intent-to-treat samples will be used to analyze data. The choice of statistical approach depends on the amount of missing data at post- and follow-up-assessment. In the case of less than 12% missing data, the Last Observation Carried Forward (LOCF) method will be applied to estimate effects. Then, the primary outcome will be computed with an analysis of covariance (ANCOVA), using the pre-scores as a covariate and the post-scores as the dependent variable.

If more than 12% of participants stop the treatment, we will use linear mixed models. This method is recommended for intent-to-treat-analyses with a high amount of dropouts due to its potential to reduce bias caused by missing data. The level of significance will be 5% (α = 0.05). Significance testing of the diagnostic status and clinically significant change will be conducted with chi-square tests. Regression analyses will be used to identify predictors of treatment outcome.

## Discussion

SAD is one of the most common mental disorders, causing personal suffering, reduced quality of life and work-related problems to a multitude of people worldwide. Without adequate treatment, SAD remains a chronic condition. The development of easily accessible, effective and flexible treatments is an important challenge to current research because the majority of those in need do not receive qualified treatment. Technologies such as the Internet open up new possibilities for low-threshold interventions. Internet-based treatments have become an increasing field of research with programs targeting several mental disorders, and SAD in particular. Several studies have demonstrated the efficacy of this new form of psychological intervention and as a consequence, countries such as Great Britain, Sweden and the Netherlands have integrated Internet-based interventions into regular health care.

With this study, we hope to gain further insight in the efficacy of web-based self-help for SAD. Especially, we are interested in the additional value of group guided support. Whereas previous studies have shown promising results of web-based interventions regarding the reduction of social phobic symptoms, the question of how much support and what kind of support is needed during the treatment still remains unresolved. Inspired by traditional, effective group treatments for SAD, the integration of communication forums in an internet-based intervention yields several advantages: Communication within the group can enable processes that have been well established in face-to-face group therapy, such as mutual support, motivation and learning from others. For instance, learning about the other participants’ experiences can motivate patients to face new challenges. Regarding economic aspects, the group format can maximize cost-effectiveness by providing a treatment to several patients at once, hereby reducing therapist hours. In a grander scheme, positive results of this study would further establish the efficacy of internet-based treatments and lay groundwork for the development of group guided self-help treatments for other disorders than SAD.

Furthermore, we hope not only to be able to demonstrate the efficacy of peer-supported Internet-based treatments, but also to identify potential moderators and mediators of outcome. Knowledge on mediators and moderators of treatment success will help to understand the processes involved in internet-based interventions and eventually facilitate selective indication.

There are, however, limitations of the trial. First, the sample will be recruited from the community via our website and internet forums. One can argue that this kind of self-selected sample does not represent the general population. One critical aspect is whether Internet literacy prevails in the general population. Statistics show that, in fact, it does: Internet use is increasingly common, with for example 85% of the Swiss population using the Internet (Bundesamt für Statistik http://www.bfs.admin.ch/bfs/portal/de/index/themen/16/04/key/approche_globale.indicator.30106.301.html?open=1#1). Regarding demographic and epidemiologic characteristics, it has been shown that patients undergoing web-based treatments resemble national samples and have disorders as severe as those undergoing traditional psychotherapy [45].

The other main concern is that the results might be biased because the self-selected sample is especially motivated for an internet-based treatment. This argument cannot be as easily dissipated. Further research has to show whether and how the findings can be generalized to samples in regular psychiatric settings. In conclusion, with this study we aim to provide insight into new possibilities to improve Internet-based treatments for SAD in terms of efficacy, adherence and costs.

## Trial status

Trial start date: November 11^th^ 2013.

Currently recruiting (N_current_ = 83 as of April 10^th^ 2014).

## Competing interests

The authors declare that they have no competing interests.

## Authors’ contributions

TB obtained funding for this study. TB and AS contributed to the study design. AS drafted the manuscript. All authors contributed to the further writing of the manuscript. All authors read and approved the final manuscript.

## Pre-publication history

The pre-publication history for this paper can be accessed here:

http://www.biomedcentral.com/1471-244X/14/115/prepub
